# Notes on Post-Partum Hemorrhage

**Published:** 1881-02-20

**Authors:** F. E. Bodemann

**Affiliations:** Detroit, Mich.


### NOTES ON POST PARTUM HÆMORRHAGE.

BY F. E. BODEMANN, M. D., DETROIT, MICH.

While a student in the University College, London, England, in the summer of 1875, I was the only student on the obstetrical list; usually there are from five to ten. I had a very great amount of work to perform, as the district surrounding the hospital was large and exceedingly populous, over five thousand births occurring annually in the maternity department. Having sometimes to attend to as many as three births in a single night, I was frequently fatigued. One night I was particularly so, a night which I shall never forget. I was called to a patient between 1 and 2 o'clock a. m., about a mile distant from the hospital. On my arrival, the os was found undilated, pains not severe and infrequent; multipara. She complained of having a uterine tumor of some kind. After having given her a full dose of opium, I concluded I could safely leave her until morning, thinking I could have a few hours of rest. In an hour from that time I was again called by the nurse, but being exceedingly sleepy thought I could delay calling for half an hour, and consequently overslept. Again I was called the third time, and went in haste; found the child born, washed, dressed, cord cut and tied, and the state of the mother alarming. The placenta was still in the uterus and blood gushing from it, and patient almost completely exsanguinated. The extremites were cold, pulse imperceptible, respiration stertorous, forehead covered with cold clammy sweat, mattrass soaked with blood, also blood on the floor; in fact it was one of the worst cases of post partum hæmorrhage I ever saw. Visions of passing from ten to twenty years in Newgate prison for manslaughter passed through my mind, as the laws of England are exceedingly strict in these matters. Without waiting to oil my hand, I introduced it immediately into the uterus and removing the placenta flung it on the floor. I gave one-half fluid ounce of ergot in combination with ammonia and hot brandy to facilitate its absorption. I also lowered the head, and elevated the limbs above the body, and pressed the blood backward with my hands, tore up a sheet and put a bandage tightly around the cephalic extremities, and introducing one hand into the uterus, I placed the other on the abdomen. If I had had a syringe I would have injected chloride of iron into the uterine cavity, according to the method of Dr. Robert Barnes. Had I been possessed of a hypodermic syringe I would have injected ergotine into a vein, but all these were wanting, so I had to do the best I could under the circumstances. The woman's life was saved, but she came nearer going than I care to have another go under like circumstances. She had severe cephalalgia for days afterwards, but small doses of quinine and big doses of Quevenne's iron, beef-tea, raw eggs with cognac soon restored her to her normal state.

Had I lost that woman I should to-day consider myself guilty of manslaughter, and whether prosecuted or not, it would have caused me many troubled thoughts. I do not endeavor to exculpate or exonorate myself, but it taught me a most impressive lesson, which I can never forget. No law in England or the United States can compel us to attend a case, but remember when you enter the room of the parturient woman, you are responsible to your own sense of morality and honor to that patient, and to the laws of your fellowmen and country. So be careful, fellow students and practitioners, how you assume that grave responsibility, and furthermore, if you lose that patient through carelessness, ignorance or neglect, you are morally if not legally guilty of murder.

A graduated homœopath, in this city, attended a case of a primipara and made no effort to, nor did he make, a vaginal examination. How would this resulted to mother and child had there been a transverse presentation? Mr. Christopher Heath, one day coming late to his clinic, observed: "I have been to Norwich to give testimony in a case where an inebriated medical man cut off three or four feet of intestine for umbilical cord, and then perceiving his mistake, told the nurse to burn it. This excited her suspicions. He was arrested, tried and acquitted, and then, rejoicing in his notoriety and celebrity, opened the public houses and hired a brass band, all of which so disgusted the decent citizens that he was re-arrested and got four month ."

In the Detroit *Review of Medicine* of August, 1876, it is reported of a quack, who used a bucket-bail, ruptured the uterus and jerked the under jaw off the fœtus, and was sentenced to ten days imprisonment; during that time he sold more medicine than all the doctors in the village.

Some doubt the possibility of the existence of concealed hæmorrhage. I was called to a pluripara; all went normally, and there was only ordinary oozing from the vagina. I was about to put on the binder, (such is the English rule), when I noticed that the flaccid abdomen began to enlarge. I pressed my hand on it and out gushed a stream of blood. For experiment, I took my hand off and again it became extended, though the amount of blood escaping from the vagina was so small that no one would call it post partum hæmorrhage. And again, on pressing, the same was repeated. I then gave ergot and applied cold, and the uterus soon became hard and firmly contracted. It is the custom of Dr. Graily Hewitt, of the University College Hospital, London, to have students give ergot after parturition to lessen the possibility of post partum hæmorrhage. This is on the theory of clots causing after pains, and as ergot expels these clots, the pain diminishes.

In the short space of six months, during which I attended cases in his department, I had one forceps case, several still-born, three breech, one child with six digits, one with club-foot, and saw, but was not allowed to examine, a partial placenta-prævia, and saw the professor perform six ovariotomies. The doctor, unlike many Englishmen, is kind, courteous, polite and obliging to Americans.

The above are a few notes from hospital life in the great city of London with its 3,600,000 inhabitants.—*Michigan Medical News.*

